# New insights into the role of mast cells as a therapeutic target in cancer through the blockade of immune checkpoint inhibitors

**DOI:** 10.3389/fmed.2024.1373230

**Published:** 2024-02-28

**Authors:** Domenico Ribatti

**Affiliations:** Department of Translational Biomedicine and Neuroscience, University of Bari Medical School, Bari, Italy

**Keywords:** immune checkpoint inhibitors, PD-1/PDL-1, tumor growth, mast cells, tumor therapy

## Abstract

Mast cells release different anti-and pro-inflammatory agents changing their role from protective to pro-inflammatory cells involved in the progression of different pathological conditions, including autoimmune diseases and tumors. Different mediators released by mast cells are involved in their biological activities which may be anti-tumorigenic and/or pro-tumorigenic. For these reasons, tumor mast cells have been considered a novel therapeutic target to prevent tumor progression and metastatic process. Many different agents have been suggested and used in the past pre-clinical and clinical settings. Among the novel immunotherapeutic approaches to cancer treatment, different immune checkpoint inhibitors targeting PD-1/PDL-1 have been used in the treatment of many human tumors improving overall survival. In this context, inhibition of mast cell activity may be considered a novel strategy to improve the efficacy of anti-PD-1/PDL-1 therapy. The blockade of the PD-1/PD-L1 interaction may be suggested as a useful and novel therapeutic approach in the treatment of tumors in which mast cells are involved.

## Tumor mast cells and related therapeutic approaches

Mast cells have multiple roles extending beyond their classical role in Ig-E-mediated allergic reactions. Mast cells release different anti-and pro-inflammatory agents changing their role from protective to pro-inflammatory cells involved in the progression of different pathological conditions, including autoimmune diseases and tumors (Table 1). Mast cells can be recruited into the tumor microenvironment by different chemotactic molecules released by tumor cells. One of the main chemoattractant factors produced by tumor cells is stem cell factor (SCF), which recruits mast cells expressing its tyrosine kinase receptor c-kit (CD117). Mast cells can exert both anti-tumorigenic and/or pro-tumorigenic roles (Table 2). Mast cells may exert detrimental effects on the host by releasing cytokines and growth factors, such as fibroblast growth factor2- (FGF-2), vascular endothelial growth factor (VEGF), nerve growth factor (NGF), and interleukin-8 (IL-8), which stimulate tumor cell expansion. Mast cells are a major source of histamine, which can induce tumor cell proliferation through H1 receptors while suppressing the immune system through H2 receptors. Mast cells produce several angiogenic factors, as well as proteases, which promote tumor vascularization and tumor invasiveness, respectively. By contrast, mast cells may promote the inhibition of tumor cell growth, tumor cell apoptosis, and inflammation.

**Table 1:** Different types of cancer in which mast cells are involved.

**Head and neck**
Oral squamous carcinoma (1)
Localization/Prognosis (Intratumoral-Good prognosis)
**Gastro-intestinal tumors**
Esophageal carcinoma (2)
Localization/Prognosis (Intratumoral-Bad prognosis)
Gastric cancer (3)
Localization/Prognosis (Intratumoral-Bad prognosis)
Colorectal cancer (4)
Localization/Prognosis (Peritumoral-Bad prognosis)
Cholangicarcionoma (5)
Localization/Prognosis (Intratumoral-Bad prognosis)
Pancreatic cancer (6)
Localization/Prognosis (Intratumoral-Bad prognosis)
**Genito-urinary tract**
Clear cell renal carcinoma (7)
Localization/Prognosis (Peritumoral-Bad prognosis)
Prostate cancer (8)
Localization/Prognosis (Intratumoral-Bad prognosis)
Endometrial cancer (9)
Localization/Prognosis (Intratumoral-Bad prognosis)
**Skin**
Melanoma (10)
Localization/Prognosis (Peritumoral-Bad prognosis)
Mastocytosis (11)
Localization/Prognosis (Peritumoral-Bad prognosis)
Breast cancer (12)
Localization/Prognosis (Tumor stroma-Bad prognosis)
**Respiratory tract**
Laryngeal carcinoma (13)
Localization/Prognosis (Peritumoral-Bad prognosis)
Lung cancer (14)
Localization/Prognosis (Intratumoral-Bad prognosis)
**Hematological tumors**
Multiple myeloma (15)
Localization/Prognosis (Intratumoral-Bad prognosis)
Chronic lymphocytic leukemia (16)
Localization/Prognosis (Intratumoral-Bad prognosis)
Myelodysplastic syndrome (17)
Localization/Prognosis (Intratumoral-Bad prognosis)

**Table 2:** Mediators released by mast cells able to stimulate or inhibit tumor growth.

**Stimulators**
Cytokines and growth factors
Fibroblast growth factor-2 (FGF-2) (18)
Vascular endothelial growth factor (VEGF) (19)
Nerve growth factor (NGF) (20)
Interleukin-8 and 10/high expression (IL-8, IL-10) (21)
Bioactive monoamines
Histamine (H1 receptors) (22)
Proteases
Tryptase, Chymase (23)
Matrix metalloproteinase (MMPs)-2 and MMP-9 (24).
**Inhibitors**
Cytokines and growth factors
Tumor necrosis factor alpha (TNFα) (25)
Interferon alpha (IFNα) (25)
Transforming growth factor beta (TGF-β) (26).
IL-1, IL-2, IL-4, IL-6, IL-10/low expression (21)
Bioactive monoamines
Histamine (H2 receptors) (22)
Proteases
Tryptase by activating protease-activated receptors (PAR-1and-2) (25)

by releasing cytokines such as inreleukin-1 (IL-1), IL-4, IL-6, and tumor necrosis factor alpha (TNF-α). Chondroitin sulfate may inhibit tumor cell diffusion and tryptase causes both tumor cell disruption and inflammation through activation of protease-activated receptors (PAR-1 and -2). Two mast cell phenotypes have been described called mast cell 1 and 2 (MC1 and MC2), related to pro-inflammatory and anti-inflammatory profiles, respectively. Mast cells promote tumor development by alterations in stroma-epithelial interactions, by inducing tumor angiogenesis and lymphangiogenesis, and by releasing different cytokines and growth factors. In solid and hematologic tumors, mast cells may be localized in intra-tumoral or peri-tumoral areas, with expression of favorable/unfavorable and, respectively, bed prognosis (25).

Based on the involvement of mast cells in tumor growth, these cells have been recently considered a novel therapeutic target in the control of tumor progression and metastatic capability. Many different agents have been suggested and used in pre-clinical and clinical settings. These therapeutic agents include inhibitors of c-kit (imatinib mesylate, mastinib, nilotinib, dasatinib, sunitinib, midostaurin, and ibrutinib). In this context, imatinib mesylate (Gleevec), which exerts inhibitory activity against the signaling cascade activated by CD117 (27), has been used against gastrointestinal stromal tumors (GIST) and metastatic melanoma with c-Kit mutations (28, 29). Masitinib has been used in the treatment of mastocytosis, GIST, colon cancer, prostate cancer, and pancreatic cancer (30). Sunitinib has been used in patients with imatinib-resistant GIST (31). Gabexate mesylate an inhibitor of tryptase has been used as an inhibitor of colon cancer growth with an anti-angiogenic effect (32). Cromolyn sodium, a mast cell stabilizing agent (33) that prevent cell degranulation (34), has been used in a xenograft mouse model of thyroid cancer (35). Obatoclax, which binds and blocks the anti-apoptotic activity of members of the Bcl-2 family, induces growth arrest in human neoplastic mast cells, and different mast cell lines (36), and exerts synergistic antineoplastic effects when combined with dasatinib (36).

H1 receptor antagonists reduced tumor growth, mast cell infiltration, and VEGF levels through the inhibition of hypoxia-inducible factor-1alpha (HIF-1α) in melanoma-bearing mice (37). Moreover, treatment with cimetidine, an H2 receptor antagonist, slows the growth of tumors in mice (38, 39). Chondroitin sulfate may inhibit tumor growth cell diffusion through activation of PAR-1 and-2 (40).

## Immune checkpoint inhibitors

Different studies have highlighted the importance of the programmed cell death-1 (PD-1)/programmed cell death ligand-1 (PD-L1) pathway controlling inflammation degree to prevent an exacerbated immune response in tumor growth, in which PD-L1 expressed on tumor cells can inhibit the effector functions of CD8^+^ T cells, leading to the progression of tumors (41). Different immune checkpoint inhibitors targeting PD-1/PDL-1 have been used in the treatment of many human tumors, such as melanoma, non-small-cell lung cancer, and renal cancer, improving overall survival (42, 43). However, this therapeutic approach may be ineffective, because of the development of resistance mechanisms mediated by inflammatory cells present in the tumor microenvironment, including mast cells.

## Relationship between tumor-infiltrating mast cells and response to anti-PD1/PD-L1 blockade

Human mast cells express several co-stimulatory and co-inhibitory molecules, including PDL-1 and PD-L2 (44). In the skin, mast cells express high levels of PD-L1, and in contact hypersensitivity mast cell absence abolished the PD-L1 blockade effect on CD8^+^T-cell activation (45). According, high levels of PDL-1 in mast cells promotes T cell immunosuppression and tumor growth in gastric cancer (46).

In high-grade serous ovarian cancer, infiltration of mast cells is associated with a decreased response to anti-PD1 blockade (47). Similarly, in a melanoma experimental model of resistance to anti-PD-1 therapy, high infiltration of mast cells predicted poor response to anti-PD1 blockade (48). An increased number of mast cells was detectable in melanoma patients after anti-PD1 therapy (49). In tumor histological sections, a co-localization of mast cells and forkhead box P3 (FOXP3)-positive Treg cells have been recognizable and associated with a down-modulation of HLA class I on tumor cells and correlated with resistance to anti-PD-1 therapy. Melanoma cells secrete chemokine (C-X-C motif) ligand 10 (CXCL10) that binds CXC motif chemokine receptor 3 (CXCR3) expressed by mast cells, favoring the recruitment of mast cells (49). Anti-PD1 treatment activates and induces expression on mast cells leading to therapeutic resistance through stimulation of angiogenesis and tumor growth (50).

Otherwise, the reduction of mast cells is associated with an improvement in the efficacy of anti-PD-1/anti-PD-L1 blockade. Combining anti-PD-1 with sunitinib or imatinib, but not PD-1 blockade alone, resulted in the depletion of mast cells and tumor regression (48). Cromolyn sodium decreases mast cell infiltration, the release of inflammatory cytokines, and improves the efficacy of anti-PD1 therapy (50). Targeting mast cells with ketotifen enhances T cells’ infiltration and cytotoxic capacity and sensitizes sarcoma cells to anti-PDL-1 therapy (51).

## Concluding remarks

Mast cells play a crucial role in the control of tumor immunity and tumor growth. They can modulate the biological activity of immune and non-immune components of the tumor microenvironment through the release of a plethora of mediators, leading to different cancer-promoting and cancer-suppressive activities. The reduction of mast cell infiltration may be considered a novel therapeutic approach to cancer treatment. Mast cells can be therapeutically targeted by decreasing their number through c-Kit inhibitors; modulating mast cell activation and phenotype, and altering secreted mast cell mediators. In this context, inhibition of mast cell activity may be considered a novel strategy to improve the efficacy of anti-PD-1/PDL-1 therapy. The blockade of the PD-1/PD-L1 interaction may be suggested as a useful and novel therapeutic approach in the treatment of tumors in which mast cells are involved.

## Author contributions

DR: Writing – original draft, Writing – review & editing.
